# Human Liver Regeneration: An Etiology Dependent Process

**DOI:** 10.3390/ijms20092332

**Published:** 2019-05-10

**Authors:** Matthias Van Haele, Janne Snoeck, Tania Roskams

**Affiliations:** Department of Imaging and Pathology, Translational Cell and Tissue Research, KU Leuven and University Hospitals Leuven, 3000 Leuven, Belgium; janne.snoeck@student.kuleuven.be (J.S.); tania.roskams@kuleuven.be (T.R.)

**Keywords:** liver regeneration, liver progenitor cell, ductular reaction, human liver diseases, acute liver damage, chronic liver damage, liver carcinogenesis

## Abstract

Regeneration of the liver has been an interesting and well-investigated topic for many decades. This etiology and time-dependent mechanism has proven to be extremely challenging to investigate, certainly in human diseases. A reason for this challenge is found in the numerous interactions of different cell components, of which some are even only temporarily present (e.g., inflammatory cells). To orchestrate regeneration of the epithelial cells, their interaction with the non-epithelial components is of utmost importance. Hepatocytes, cholangiocytes, liver progenitor cells, and peribiliary glands have proven to be compartments of regeneration. The ductular reaction is a common denominator in virtually all liver diseases; however, it is predominantly found in late-stage hepatic and biliary diseases. Ductular reaction is an intriguing example of interplay between epithelial and non-epithelial cells and encompasses bipotential liver progenitor cells which are able to compensate for the loss of the exhausted hepatocytes and cholangiocytes in biliary and hepatocytic liver diseases. In this manuscript, we focus on the etiology-specific damage that is observed in different human diseases and how the liver regulates the regenerative response in an acute and chronic setting. Furthermore, we describe the importance of morphological keynotes in different etiologies and how spatial information is of relevance for every basic and translational research of liver regeneration.

## 1. Introduction

**Figure 1:**
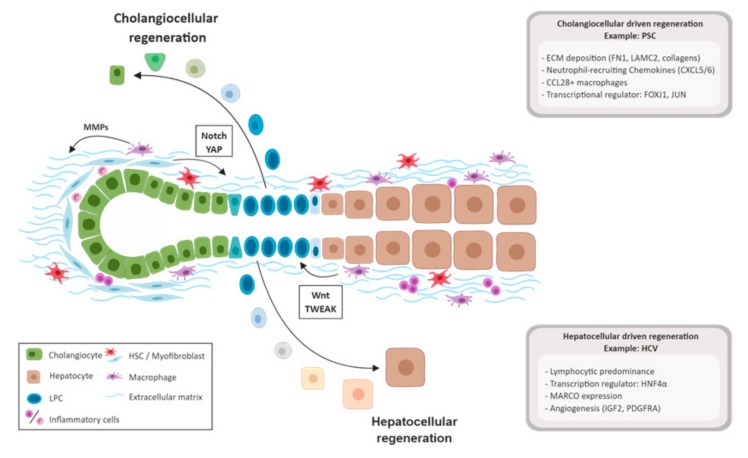
Overview of liver regeneration in chronic hepatic diseases. Activation of progenitor cells is induced by senescence or exhaustion of cholangiocytes or hepatocytes. After longstanding hepatocellular damage, macrophages stimulate the proliferation of liver progenitor cells (LPCs) via the Wnt and TWEAK pathway. In chronic cholangiocytic damage, myofibroblasts influence LPCs via the Notch and YAP pathway. In addition, HSC, myofibroblasts, and macrophages regulate the extracellular matrix (ECM) through deposition of collagens and secretion of matrix metalloproteases (MMPs). Abbreviations: yes-associated protein 1 (YAP), TNF-related weak inducer of apoptosis (TWEAK), hepatic C virus (HCV), hepatocyte nuclear factor 4 alfa (HNF4α), macrophage receptor with collagenous structure (MARCO), insulin-like growth factor 2 (IGF2), platelet-derived growth factor receptor A (PDGFRA), primary sclerosing cholangitis (PSC), fibronectin (FN1), laminin subunit gamma-2 (LAMC2), C-X-C motif chemokine ligand 5 and 6 (CXCL5/6), chemokine (C-C motif) ligand 28 (CCL28), forkhead box protein J1 (FOXJ1), and jun proto-oncogene (JUN).

To fully grasp the function of the human liver, a minimal anatomical and histological knowledge is required. The liver is the second largest organ divided into two lobules (right and left). Furthermore, subdivision in segments is based upon the vascular flow and bile duct organization (the Couinaud classification) [1]. On a microscopical level, the concept of a hepatic lobule with a triangular shape was introduced. In this model, the base is aligned with the portal triads and a central vein at the apex of the triangle. The portal triad contains a portal vein, a hepatic artery, and an intrahepatic bile duct. Both vessels in the portal triad supply blood from the gut and the lower part of the body to the liver, while the bile flows in the opposite direction towards the extrahepatic bile duct. In between the portal triad and central vein, hepatocytes are present. These are the main epithelial component of the liver, in addition to cholangiocytes. The hepatocytes are ordered in singular cords from the portal triad towards the central vein. Interestingly, the hepatocytes located near the portal tract perform different functions than those in the middle and those around the central vein [2]. In addition, the oxygen level of the blood around the portal tract is higher compared to the level at the central vein. This unbalanced distribution results in a more ischemic vulnerability of the hepatocytes in the neighborhood of the central vein. The cholangiocytes, on the other hand, line the bile duct, the bile ductule, and partially the canal of Hering [3]. The canal has been described as the transition zone between the hepatocytes and the biliary tree (Figure 1). This interesting anatomical transition zone is known to harbor the liver progenitor cells (LPCs). The progenitor cells are small epithelial cells with an oval nucleus and scanty cytoplasm [4]. Starting at the periportal area, a ductular reaction is observed in almost all acute and chronic liver diseases [3]. This reaction has a ductular morphology but not necessarily a ductular origin. Moreover, the ductular reaction may derive from proliferating cholangiocytes, differentiating LPCs or de-differentiated hepatocytes. To date, the specific origin of the ductular reaction is unresolved, since lineage tracing in humans is not feasible. Non-epithelial cell components of the liver are the liver sinusoidal endothelial cells (LSECs), hepatic stellate cells (HSCs), and subtypes of inflammatory cells. Kupffer cells are the resident macrophages of the liver and are located within the liver sinusoids [5]. This anatomical extraordinary machinery allows the liver to exercise a great variety of functions: Removal of metabolic products, bile production, bile circulation, glucose metabolism, synthesis of proteins, immunological defense, and many more.

As one can imagine, this extensive variety of functions allows an opportunity to multiple ways of failure, each of which can result in a particular liver disease. Despite the long list of potential diseases, the liver is often able to compensate for the damage due to its enormous regenerative capacity. Liver regeneration is exceptionally well orchestrated, surprisingly fast, and often without noticeable clinical symptoms [6]. In addition, the total liver volume strives to be continuously stable, which seems to be required for homeostasis of the body [7]. Intriguingly, this capacity to regenerate is only triggered in the presence of damage, as the liver is a quiescent organ with a low turnover time under normal circumstances [6]. Regeneration originates from the epithelial cell compartment. However, interaction with its niche, the non-epithelial cells and matrix proteins, is critical for a well-coordinated process. In this review, we aspire to give an overview of how liver regeneration is organized in humans.

## 2. Regeneration after Resection

Most knowledge regarding liver regeneration originates from rodent models in which two-thirds partial hepatectomy is performed. In this model, the residual lobes restore the surgically removed volume of the liver within one week [8]. During the regeneration, hypertrophy of the hepatocytes is the first response, followed by proliferation of the non-epithelial compartment (hyperplasia) [9,10]. However, the resected lobes never grow back. The best comparison of this model in humans is a partial hepatectomy. The most common indication for partial hepatectomy is resection of a primary or secondary liver tumor [11]. Among primary liver cancer (PLC), hepatocellular carcinoma (HCC) is the most common, followed by intrahepatic cholangiocarcinoma (ICC) [12]. Additionally, the liver is a typical site for metastasis of other tumors, mostly of gastrointestinal origin [13]. During hepatectomy, as the volume of liver remnants decreases, the risk of critical liver failure increases [14]. Therefore, resection in patients with an already impaired liver function (e.g., viral induced hepatitis) is challenging and sometimes even not feasible. An elegant technique for compensating this issue is preoperative portal vein ablation [15]. During this procedure, clotting is induced at the side of the liver were resection is needed. The clotting causes the development of hepatocytic hyperplasia at the nondiseased side. The induced hyperplasia will increase the remaining liver volume before hepatectomy that results in a better prognostic outcome of the patient [16]. The regeneration of the liver is influenced by many factors. Restoration of an intact blood flow is crucial for liver regeneration [17]. Another important determinant of regeneration after hepatectomy is the use of post-resection treatments. Some of these treatments target the blood supply of the tumor and can therefore also hamper the regenerative potential of the remaining part of the liver, since these therapies are often nonspecific [18]. Other crucial factors involved in the stimulatory effect of the regenerative response during a hepatectomy include bile acids, tumor necrosis factor-alpha, epidermal growth factor, hepatocyte growth factor, transforming growth factor alpha, insulin, and interleukin-6 [19]. In agreement with the regulatory role of the liver macrophages, most of these stimulatory cytokines and growth factors are mainly secreted by macrophages. By contrast, the amount of steatosis is recognized as a negative influence on the outcome of the patient [20]. Regeneration in humans after hepatectomy can also be seen after living donor transplantation. This procedure was implemented due to the scarcity of donor organs, in which a part of the liver of an adult is transplanted to another child or adult [21]. Interestingly, two studies observed that regeneration in terms of liver volume was most significant during the first two weeks after transplantation, and the approximated maximal liver volume was already reached after only two months [22,23]. Of interest, the recipients’ liver regenerated faster compared to the donor’s liver.

## 3. Liver Regeneration in the Diseased Liver

### 3.1. Acute Liver Damage and Regeneration

In a clinical setting, acute liver damage is referred to as a damaged liver, hepatic encephalopathy, and impaired protein synthesis. Acute failure can be induced by a variety of causes, the most common being: Acetaminophen (N-acetyl-para-aminophenol; APAP) intoxication, viral infections (hepatitis A, B, and E), alcoholic hepatitis, and autoimmune hepatitis (AIH) [24]. Acetaminophen intoxication is the most common cause in Western countries. This drug-induced hepatotoxicity is a complex process due to an interplay between time- and dose-dependent interactions. Acute liver failure following intoxication is characterized by a “hepatitis” with hepatocytic cell death, preferentially in the centrilobular area of the human liver, and is often accompanied by a prominent infiltration of ceroid-laden Kupffer cells [25]. It is certain that these macrophages play an important role in the pathogenesis and therefore regeneration of acetaminophen intoxication [26]. Otherwise, acute liver failure induced by viral hepatitis is often seen in a diffusely damaged liver without a typical zonal distribution pattern. Treatment of acute liver failure patients is often supportive and requires specific treatment adjusted for the underlying cause. However, some patients progress towards a stage where spontaneous recovery of the liver is no longer achievable or suspected and transplantation becomes necessary. Generally, important factors related to a poor outcome and therefore less likely to overcome (regenerate) acute injury are the etiology of the acute liver failure, a higher patient age, and the presence of hepatic encephalopathy [27].

**Figure 2:**
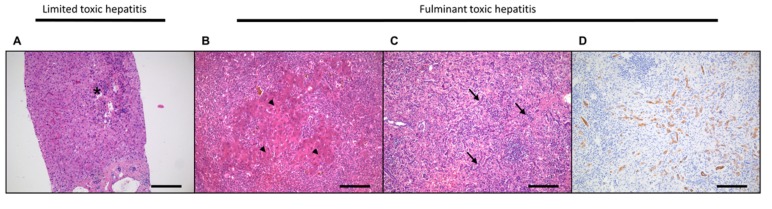
Needle biopsy of a patient with acetaminophen intoxication in the early phase. Characterized by typical centrilobular necrosis, swollen hepatocytes, and an inflammatory infiltrate (asterisk) (**A**). Explanted liver after acute acetaminophen overdose with some islands of remaining hepatocytes (arrowheads), surrounded by a fulminant inflammatory infiltrate. No ductular reaction present yet (**B**). Explanted liver after acute and chronic acetaminophen abuse with no remaining hepatocytes left. Complete collapse of the supporting framework. A prominent ductular reaction (arrows) tries to compensate for the loss of hepatocytes as a result of severe intoxication (**C**). Keratin-19 staining of the ductular reaction in a fulminant necrotizing toxic hepatitis (**D**). Original magnification ×20. Scale bar: 50 µm.

The initial regenerative response induced in the setting of acute liver failure is mainly provided by the remaining residual hepatocytes. Only when the injury is persistent or a great portion of the hepatocytes is injured do the LPCs in the canal of Hering start to proliferate intensively to compensate for this massive loss of hepatocytes (Figure 2). The activation of the progenitor cells is seen as ductular reaction, trying to maintain the critical functions of the liver. A study of our group using human acute liver failure specimens showed that a threshold of 50% loss of hepatocytes is associated with a significant decrease in the proliferative capability of the remaining hepatocytes [28]. This threshold was necessary to fully activate the LPC cell compartment. Activation of the ductular reaction was observed within the first week of injury. However, differentiation of the activated progenitor cells towards intermediate hepatocytes and adult hepatocytes was only noticed after at least one week of injury. These intermediate hepatocytes have an intermediate size and still express some LPC markers but gradually lose these markers during the progress of maturation [29]. These findings suggest that LPCs are activated and differentiate towards mature and functional hepatocytes. Interestingly, the Wnt/β-catenin signaling pathway seems to be of utmost importance in the hepatocellular regeneration. A study by our group showed that the Wnt/β-catenin signaling pathway is active in the ductular reaction of acute liver failure patients, in contrast to the Notch pathway [30]. In agreement, activation of the Wnt/β-catenin signaling pathway was shown during hepatocytic regeneration in mice after APAP treatment [31]. During another clinicopathological study of APAP-induced injury, patients demonstrated a significant correlation between immunohistochemical nuclear β-catenin staining and inducement of hepatocellular regeneration [32]. Of interest, a recent study by Brid et al. illustrated that hepatocellular senescence was macrophage-derived transforming growth factor–β1 (TGFβ1) ligand-dependent and distributed through paracrine factors [33]. Inhibition of TGFβ1 improved the regenerative capability to overcome acute hepatocytic injury. In addition, the influence of the inflammatory environment on the regenerative process cannot be neglected. An extensive evaluation of the immune response in human hepatic APAP injury observed a depletion of circulating monocytes and infiltration of hepatic macrophages in the necrotic areas [34]. Depletion of the monocytes was most prevalent in patients with a worse outcome. Furthermore, macrophage colony-stimulating factor (CSF1) is important in the development and recruitment of hepatic macrophages. A clinical study assessed the CSF1 levels in serum of acute liver failure patients [35]. High levels of CSF1 predicted a better outcome, while low CSF1 was a marker for deterioration. All aforementioned pathways have proven to be interesting for further clinical exploration and implementation of improving liver regeneration in the acute setting of liver diseases.

### 3.2. Hepatocellular Damage and Regeneration

Hepatocytic diseases are characterized by damage of the hepatocytic compartment. Due to the diversity of functions attributed to the hepatocyte, the disease spectrum is very broad. Most hepatocellular diseases have a chronical progression. Common chronic causes of hepatocellular damage are viral hepatitis B (HBV) and viral hepatitis C (HCV) infection [36,37]. Autoimmune hepatitis is another relative frequent disease in which mainly the hepatocytes are chronically damaged [38]. Due to the increasing obesity worldwide, non-alcoholic fatty liver disease (NAFLD) is rising continuously. NAFLD comprises non-alcoholic fatty liver (NAFL) and non-alcoholic steatohepatitis (NASH) [39]. NAFL and NASH show both accumulation of fat (steatosis), whereas NASH shows an additional inflammatory component. NAFLD shows many clinicopathological overlapping signs with alcoholic liver disease (ALD), such as inflammation, hepatocytic damage, steatosis, and fibrosis, leading to cirrhosis in the end stage [40]. Excessive alcohol abuse is by definition not included in NAFLD. As one can imagine, the distinction between NAFLD and ALD is not always easily made from a clinical perspective. Nevertheless, satellitosis in which ballooned hepatocytes are surrounded by neutrophilic granulocytes is a histological feature not observed in NAFLD [41]. Therefore, satellitosis is a pathological sign of active alcohol consumption.

**Figure 3:**
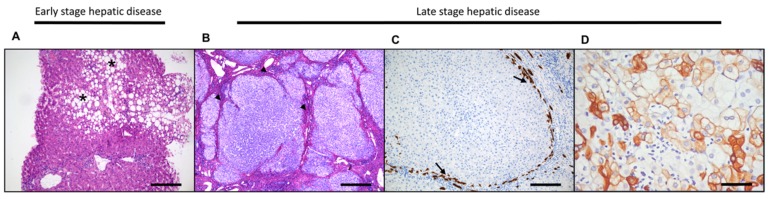
Needle biopsy of a patient with early non-alcoholic fatty liver disease (NAFLD) is characterized by macrovesicular steatosis and lobular inflammation (asterisk) in the centrilobular area (**A**). Sirius Red staining, highlighting the fibrosis in an explanted liver of a NAFLD patient, illustrates the cirrhotic end-stage of the disease by the presence of regenerative nodules of hepatocytes surrounded by bridging fibrosis (arrowheads) (**B**). Keratin-19 staining in a cirrhotic liver highlights the ductular reaction at the border of a nodule (arrow) (**C**). Keratin-7 staining in a cirrhotic liver illustrates the presence of intermediate hepatocytes and mature hepatocytes by gradual expression of CK7 (**D**). Original magnification ×20, scale bar 50 µm (**A** and **C**); ×10 original magnification, scale bar 200 µm (**B**) and ×40 original magnification, scale bar 20 µm (**D**).

All aforementioned hepatocellular diseases have in common that the hepatocytes are the main damaged cell compartment. During the primary stages of these diseases, regeneration is provided by hepatocytes [42]. However, when the hepatocytic compartment is depleted/senescent, LPCs are activated and differentiate towards hepatocytes [43,44,45]. The commitment of the progenitor cells towards the hepatocytic lineage is suggested as differentiation of LPC towards intermediate hepatocytes that are mainly located in the periportal area (Figure 3). In human diseases, intermediate hepatocytes are solely observed by immunohistochemical and morphological analysis. Therefore, it is difficult to claim that these observed intermediate hepatocytes are originating from original LPCs since there is no current technique that allows for a more robust approach. In rodents, for example, different subgroups of hepatocytes with various expression profiles and morphological locations have been identified as the source of the regeneration [46]. This variety of different subgroups of hepatocytes in rodents highlights the complexity of liver repair mechanisms. However, during the last few years, several studies have confirmed the existence and involvement of these LPCs in hepatocellular regeneration [45,47,48,49,50]. Interestingly, two studies observed a significant correlation between the ductular reaction and the stage of fibrosis in NASH patients [51,52]. Presence of the ductular reaction was recognized as a predictor of increasing fibrosis and disease activity. Additionally, the degree of ductular reaction was directly correlated with the extent of hepatocyte replicative arrest and amount of intermediate hepatocytes [53]. Similar findings were found in HCV and alcohol-induced hepatocellular damage (ALD) [53,54,55]. HSCs are important initiators and controllers of this hepatic fibrosis and therefore influence LPCs [56]. Another study confirmed this close relationship and the important interplay between HSCs and LPCs [57]—for example, through the hepatocyte growth factor (HGF) pathway, which promotes hepatocytic regeneration [58,59,60]. By contrast, crosstalk between hepatic stellate cells has been shown to stimulate the LPC proliferation towards biliary regeneration via the Notch pathway [61]. In addition, myofibroblasts are considered another import key player in the process of regeneration [62]. This regeneration-induced process is injury-dependent, in which HSC seems to be the main source of myofibroblasts. A study of Cassiman et al. elucidated different subtypes of myofibroblasts (e.g., portal/septal, interface, and perisinusoidal) by morphological and expression analysis [63]. Moreover, it is indisputable that the continuously changing interactions between the LPCs and its niche are of crucial importance in the progression of the diseases and the regeneration of the liver. Gadd et al. underlined the importance of the inflammatory infiltrate in the periductular area in NAFLD patients, in particular, the early presence of the macrophages [64]. Furthermore, macrophages are known to influence the differentiation of LPC towards hepatocytes during hepatocellular injury via the Wnt and Hedgehog pathways [61,65]. Moreover, the TNF-like weak inducer of apoptosis (TWEAK) pathway is suggested to be an inducer of the LPC in chronic hepatic C patients [66]. Of interest, another regulator of the LPCs is the autonomic nervous system [67,68]. In agreement, HSCs require synaptic neurotransmitters to promote hepatic fibrosis [69]. For example, amphiregulin is upregulated in severe NASH patients and known to induce fibrosis [70]. A recent study of Govaere et al. explored the differential gene expression of the ductular reaction between HCV and primary sclerosing cholangitis (PSC) patients [71]. This expression pattern was demonstrated to be very etiology-dependent. A strong biliary related pattern with a predominant neutrophil-attractant pattern was observed in the ductular reaction of PSC patients. While the HCV patients presented an expression of hepatocellular relating genes and a lymphocytic infiltrate, different macrophage patterns and discrepancies in extracellular matrix (ECM) deposition were observed. For example, HCV patients clearly expressed less ECM-related genes like FN1, LAMC2, and multiple collagens compared to PSC patients. Moreover, macrophages around the ductular reaction in HCV were strongly positive for macrophage receptor with collagenous structure (MARCO), while a high expression of chemokine (C-C motif) ligand 28 (CCL28), a neutrophil-chemoattractant was observed in macrophages of the portal tract. Thus, we can conclude that regeneration in all chronic hepatocellular liver diseases shows signs of overlap, but certainly also important etiology-related discrepancies.

### 3.3. Biliary Damage and Regeneration

Next to hepatocytic diseases, there are liver diseases in which the biliary tree is progressively destroyed over time, leading to cholestasis and hepatic failure in the end-stage. PSC and primary biliary cholangitis (PBC) are the two most common biliary diseases. Nevertheless, little is known about the pathophysiology of these diseases. PSC predominantly affects the larger intra- and extrahepatic bile ducts [72]. In a small group of PSC patients, no damage to the bile duct can be visualized by cholangiography. Nevertheless, biliary damage is observed in the smaller bile ducts during histological examination. This ‘small duct PSC’ variant is known to have a better prognosis but can evolve to classic PSC over time [73]. Prior to making the diagnosis of PSC, it is important to exclude causes of secondary sclerosing cholangitis, for example, choledocholithiasis or chronic bacterial cholangitis. PBC patients are characterized by damage of the smallest intrahepatic bile ducts [74]. The diagnosis of PBC is supported by detection of antimitochondrial antibodies (AMA) in the serum [75]. However, a small subpopulation is AMA negative. Liver biopsy can therefore be of crucial importance to determine the proper diagnosis, since both diseases are treated differently. Another, rarer biliary disease is the variant syndrome or ‘overlap syndrome’, in which there is an overlapping PBC and AIH component [76]. All of the described diseases can be symptomatically treated, yet no definitive treatment is available. Another, rather intriguing, disease affecting the bile ducts is the Alagille syndrome. This autosomal dominant genetic disorder is caused by a loss of function mutation in either the *JAG1* or *NOTCH2* gene [77]. This mutation results in paucity of bile ducts, leading to chronic liver failure. After procuring this mutation, research groups were able to create genetic mouse models that resemble this human disease [78,79]. Therefore, they will be able to study bile duct development and regeneration in depth.

**Figure 4:**
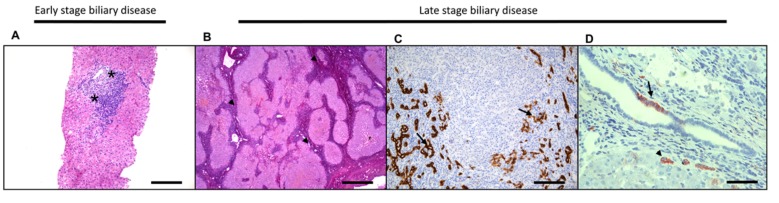
Needle biopsy of a patient with early-stage primary biliary cholangitis. This is pathologically characterized by a prominent dominant lymphocytic infiltrate and interlobular bile duct damage (asterisk) (**A**). Sirius Red staining, highlighting the fibrosis in an explanted liver of a primary sclerosing cholangitis patient. Note the distinct fibrotic “jigsaw puzzle pattern” (arrowheads) compared to hepatocytic diseases (**B**). Keratin-19 staining in a cirrhotic liver highlights the ductular reaction at the border of a nodule and in the stromal component where new biliary ducts are formed (arrows) (**C**). Neural cell adhesion molecule (NCAM) staining of the reactive bile ducts (arrowhead) and a patchy infiltrate in the mature bile ducts (**D**). Adapted from [88] with permission. Original magnification ×20, scale bar 50 µm (**A** and **C**); ×10 original magnification, scale bar 200 µm (**B**) and ×40 original magnification, scale bar 20 µm (**D**).

Biliary cells are difficult to study in human disease as they often have an overlapping expression of markers throughout the biliary tree [80]. Keratin-7 (K7) and Keratin-19 (K19) are well known typical markers of well-differentiated cholangiocytes in a normal liver. Additionally, a recent study of our group observed an intense staining of the biliary tract for Yes-associated protein 1 (YAP) and its homolog WW domain-containing transcription regulator protein 1 (WWTR1, also called TAZ) [81]. Biliary cells, similar to hepatocytes, are suspected to be able to regenerate themselves when minor and acute damage occurs. However, immunohistochemical evidence is scarce in human diseases since there are no specific markers to differentiate regenerative cholangiocytes from mature cholangiocytes. Additionally, they are in the close vicinity of the LPCs, which express overlapping markers. However, these are often distinguishable by morphology and subtle different immunohistochemical expression patterns. For example, the LPC express Sex-Determining Region Y-Box 9 (SOX9), neural cell adhesion molecule (NCAM), and epithelial cell adhesion molecule (EpCAM). Other limitations are the relatively low prevalence of these diseases, the diseases’ spectrum, and the limited amount of available tissue. Therefore, a multicenter approach to gain more insights in biliary diseases is recommended. Another interesting compartment of the biliary tree is the peribiliary gland (PBG). These glands are located in the greater extrahepatic and intrahepatic bile ducts. A study by de Jong et al. showed nicely that PBGs are capable to address biliary loss of the extrahepatic bile ducts by proliferation, differentiation, and maturation towards normal biliary epithelial cells [82]. Hence, regeneration of the extrahepatic bile duct in PSC can occur through PBG proliferation. Nonetheless, intrahepatic bile duct damage and regeneration, as seen in PBC, is suggested to be supported by the LPCs (Figure 4) [83]. However, similar to hepatocytic regeneration, rodent studies illustrate that hepatocytes and cholangiocytes can (de-)differentiate into progenitor-like cells. Another important component of this complex niche is the ECM. This dynamic microenvironment created by noncellular components provides a scaffold for the surrounding cells and is involved in cell-to-cell signaling [84]. However, in diseased liver conditions, the normal homeostasis of the ECM is disturbed, which leads to deposition of collagen type I, II, and IV over time [85]. Therefore, when biliary injury is discovered in a late stage or treatment does not help (sufficiently), fibrosis occurs due to chronic damage. In biliary diseases, fibrosis typically starts at the periportal area and extends to porto-portal septa, resulting in fibrotic nodules [86]. Typically, in biliary disease, the nodules are not nicely round-shaped but show some connections comparable to a jigsaw puzzle [87]. When fibrosis increases (long-term injury) and the normal regeneration becomes impaired, LPCs start to proliferate to compensate for the loss of cholangiocytes [83,88]. This proliferation is morphologically observed as the ductular reaction in humans [44,89]. Interestingly, in human liver specimens, it is shown that the ductular reaction in PBC and PSC cases correlates with the extent of fibrosis and clinically-relevant prognostic scores [90]. Furthermore, end-stage PBC patients were characterized by higher content of laminin around the ductular reaction compared to PSC patients. Complementary, another study demonstrated that laminin was obligatory in chronic liver diseases to maintain a good LPC-mediated regeneration [91]. Thus, there are similarities between the biliary hepatocellular regeneration. The cholangiocytes are self-sufficient for their regeneration until these cells become exhausted; at that moment, the liver progenitor cells will attempt to compensate. However, significant differences in ECM, inflammatory environment, and differentiation regulators (Figure 1) were demonstrated between hepatic and biliary ductular reaction and their niche [71]. For example, hepatocyte nuclear factor 4 alpha (HNF4α), critical for hepatic differentiation, was overexpressed and observed in LPCs of HCV patients, while on the other hand, Jun proto-oncogene (JUN) was predominantly present in PLCs of PSC patients. Thus, the mechanism has morphologically overlapping characteristics (e.g., ductular reaction), and the niches and pathways seem to be etiology-dependent.

## 4. Carcinogenesis

**Figure 5:**
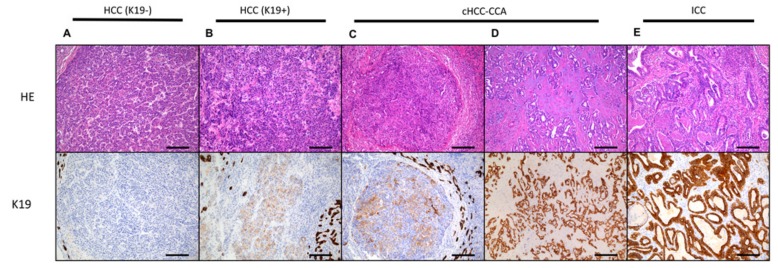
The spectrum of primary liver cancer. Firstly, the typical hepatocellular carcinoma (HCC) is observed, which is characterized by a hepatocellular morphology (trabecular growth pattern) and Keratin-19 (K19) negativity (**A**). Secondly, a carcinoma with a hepatocellular morphology and immunohistochemical expression of progenitor cell markers (e.g., K19) is noted (**B**). In the middle of the spectrum is the combined hepatocellular-cholangiocarcinoma (cHCC-CCA). This carcinoma is represented by a dual morphology and immunohistochemical expression. This tumor has a morphology that still is in accordance with hepatocellular characteristics (**C**); it also has a component with a clear cholangiocytic (glandular) growth pattern (**D**). The glandular cholangiocytic derived structures are strongly expressing K19 (**D**). At the end of the spectrum, a typical intrahepatic cholangiocarcinoma (ICC) is noted (**E**). Original magnification ×20. Scale bar: 50 µm. Abbreviation: HE, hematoxylin and eosin.

Primary liver cancer is one of the most diagnosed cancers worldwide and is currently the fifth most common cancer in men and ninth in women [92]. Liver cirrhosis is present in 80–90% of the diagnosed PLC cases and is thus a strong predictor and stimulatory environment for HCC [93]. PLC can also arise in a liver that is not cirrhotic. However, these noncirrhotic livers regularly show signs of chronic damage but were not cirrhotic yet [94]. These chronic cycles of regeneration trigger chromosomal instability and impairment of the regenerative process, which eventually leads to carcinogenesis [95]. The premalignant lesion in the liver is referred to as a dysplastic nodule [96]. Morphologically, this lesion is observed as a nodular structure of less than 2 cm with an increased cellularity, cytological atypia, and unpaired arteries. Often, the differential diagnosis between the dysplastic nodule and HCC proves difficult. Testing for the presence of heat shock protein 70 (HSP70), glypican 3, and glutamine synthetase can resolve this problem, as positivity of two out of three proteins is only observed in HCC [97,98]. However, the underlying molecular mechanisms of HCC remain undiscovered. Multiple studies found a heterogeneous mutational landscape in which telomerase reverse transcriptase (TERT) was the most common mutated gene [99,100,101]. Heterogeneity of HCC is not only observed within the molecular pathways of the tumor but also in its morphological presentation [102,103]. In addition, a broad spectrum of different subtypes is noted within the group of PLC (Figure 5) [104]. This spectrum comprises HCC with a typical hepatocellular morphology on the one side and intrahepatic cholangiocarcinoma with a typical cholangiocellular morphology on the other side. However, an intriguing group of PLC presents itself with mixed characteristics, the so-called combined (or mixed) hepatocellular-cholangiocarcinoma (cHCC-CCA). Interestingly, this subgroup expresses typical stemness, hepatocellular, and cholangiocellular markers [81,105]. A thorough genomic analysis of these tumors confirmed the existence of this distinct subgroup [106]. These findings suggest that cHCC-CCA is a subgroup within the spectrum of PLC, derived from the LPC. Arguments in favor of this hypothesis are several: Morphological, immunohistochemical, genetic, and prognostic elements that can solely be explained by the presence of progenitor/stem cell-like state at some point during carcinogenesis. In addition, we observed a group of PLC that morphologically resembles HCC but immunohistochemically expresses typical stemness markers (e.g., K19) [107,108]. This subtype of HCC is correlated with a more aggressive behavior [81]. After years of many disappointing trials in HCC, many novel therapies targeting the tumor microenvironment (TME) are arising [109]. Unfortunately, little is currently known about the TME in PLC. A recent study of Kurebayashi et al. associated the prognostic outcome of HCC patients with the composition of the immune infiltrate [110]. In conclusion, we have to recognize the heterogeneity in PLC by considering several elements: The different etiological backgrounds, the histopathological subtypes, the mutation-based subtypes, and the differences of the TME in PLC. A thorough characterization and classification will prove to be beneficial for the PLC patient in the long run.

## 5. Conclusions

The epithelial components of regeneration in the liver are hepatocytes, cholangiocytes, liver progenitor cells, and peribiliary glands. It becomes clear that regeneration in the human liver depends on which epithelial component is injured, but also which specific pathology (e.g., inflammation-induced versus metabolic) is the underlying trigger for the injury. Vast evidence shows that the surrounding microenvironment of this epithelial regenerative process is a key player in regeneration and in carcinogenesis of the human liver.

## Abbreviations

LPCs	Liver progenitor cells
LSECs	Liver sinusoidal endothelial cells
HSCs	Hepatic stellate cells
PLC	Primary liver cancer
HCC	Hepatocellular carcinoma
ICC	Intrahepatic cholangiocarcinoma
HNF4α	hepatocyte nuclear factor 4 alfa
TWEAK	TNF-related weak inducer of apoptosis
IGF2	insulin-like growth factor 2
APAP	N-acetyl-para-aminophenol
PDGFRA	Platelet-derived growth factor receptor A
FN1	Fibronectin 1
LAMC2	Laminin subunit gamma-2
CXCL5	C-X-C motif chemokine ligand 5
CXCL6	C-X-C motif chemokine ligand 6
FOXJ1	Forkhead box protein J1
JUN	Jun proto-oncogene
AIH	Autoimmune hepatitis
TGFβ1	Transforming growth factor–β1
CSF1	Colony-stimulating factor
HBV	Hepatitis B virus
HCV	Hepatitis C virus
NAFLD	Non-alcoholic fatty liver disease
NAFL	Non-alcoholic fatty liver
NASH	Non-alcoholic steatohepatitis
MARCO	Macrophage receptor with collagenous structure
CCL28	Chemokine (C-C motif) ligand 28
PSC	Primary sclerosing cholangitis
PBC	primary biliary cholangitis
AMA	Antimitochondrial antibodies
ECM	Extracellular matrix
K7	Kertin-7
K19	Keratin-19
YAP	Yes-associated protein 1
WWTR1	WW domain-containing transcription regulator protein 1
SOX9	Express Sex-Determining Region Y-Box 9
NCAM	Neural cell adhesion molecule
EpCAM	Epithelial cell adhesion molecule
PBG	Peribiliary gland
HGF	Hepatocyte growth factor
HSP70	Beat shock protein 70
TERT	Telomerase reverse transcriptase
cHCC-CCA	Combined hepatocellular-cholangiocarcinoma
